# Machine learning to predict untreated dental caries in adolescents

**DOI:** 10.1186/s12903-024-04073-4

**Published:** 2024-03-09

**Authors:** Rafael Aiello Bomfim

**Affiliations:** School of Dentistry, Federal University of Mato Grosso do Sul, Campo Grande, Brazil

**Keywords:** Dental caries, Adolescents, Machine learning, Primary health care contribution statement

## Abstract

**Objective:**

This study aimed to predict adolescents with untreated dental caries through a machine-learning approach using three different algorithms

**Methods:**

Data came from an epidemiological survey in the five largest cities in Mato Grosso do Sul, Brazil. Data on sociodemographic characteristics, consumption of unhealthy foods and behaviours (use of dental floss and toothbrushing) were collected using Sisson’s theoretical model, in 615 adolescents. For the machine learning, three different algorithms were used: (1) XGboost; (2) decision tree and (3) logistic regression. The epidemiological baseline was used to train and test predictions to detect individuals with untreated dental caries, through eight main predictor variables. Analyzes were performed using the R software (R Foundation for Statistical Computing, Vienna, Austria). The Ethics Committee approved the study..

**Results:**

For the 615 adolescents, xgboost performed better with an area under the curve (AUC) of 84% versus 81% for the decision tree algorithm. The most important variables were the use of dental floss, unhealthy food consumption, self-declared race and exposure to fluoridated water.

**Conclusions:**

Family health teams can improve the work process and use artificial intelligence mechanisms to predict adolescents with untreated dental caries, and, in this way, schedule dental appointments for the treatment of adolescents earlier.

## Introduction

The Brazilian Unified Health System (SUS) has been providing universal health coverage through the Family Health Strategy (FHS) [1] and increasing the number of primary and secondary oral health care [2]. Despite advancements in Brazilian public policy, it is challenging to set priorities on the dental agenda, especially concerning the adolescent population.

Adolescence is particularly a relevant moment for studying the use of health services and the burden of dental diseases, like dental caries [3–7]. This reinforces the importance of primary care services in organizing access to and offering comprehensive care to adolescents, especially those who have untreated caries. The need for treatment, such as caries and pain, is one of the main reasons for using dental services in adolescents [8]. Moreover, the DMFT in Brazilian 12 years old was 2,1 in 2010, with untreated dental caries representing 53% of the index. Moreover, it concentrates on the most vulnerable and socially deprived people [7].

A machine learning approach would help predict those adolescents at higher risk for tooth decay and help schedule a dental visit and establish better oral health, and consequently, a better quality of life [9–11]. This approach has not been tested using Sisson´s theoretical model, which describes the social inequalities in oral health, to select variables associated with dental caries, considering individual, behaviour and contextual predictors. However, Primary health care (PHC) workers in the Family health strategy (FHS), with a simple input of variables collected by community health workers, could predict those adolescents with untreated dental caries. It would be great to organise the dental agenda and set priorities due to the importance of oral health on general and global health [12].

Therefore, the objective of the present study was to predict adolescents with untreated dental caries using Sisson’s theoretical model. The hypothesis to be tested is that the selected variables containing individual, contextual and health behaviours variables will have a good performance, i.e., predicting correctly more than 70% of adolescents with untreated dental caries.

## Methods

This study was a population-based epidemiological survey representing the five largest cities (over 80,000 inhabitants) in Mato Grosso do Sul, Brazil. These five cities are the most representative of the four territorial macro-regions in the State (Dourados and Ponta Porã are in the same macro-region) and are the most affluent areas [13].

### Sample size

A formula was used to calculate the sample size for dental caries, which considers the values of the mean and standard deviation of the DMFT index in the Central-west region of Brazil [4, 13]. The calculated sample size was 520 schoolchildren, including denials (30%). Considering the 115 eligible schools, we had around five students per school. After accounting for 35% of school denials due to school principals unwilling to participate, seven students per school were estimated, which we opted to round up to 10 students per school.

### Oral health teams’ calibration

Five dental teams in each city, composed of a dentist and an annotator, received explanations with 32 h of practical training, based on consensus. The intra- and inter-examiner reliability test showed a Kappa coefficient of 0.73 [13].

### Main outcome

The outcome variable was based on the DMFT index, as recommended by the World Health Organization (WHO) [14]. The index measures the caries experience of 12-year-old children. We used the D – Decayed component of the index, stratified into adolescents with D ≥ 1. In the survey, caries was considered a groove, fissure or smooth surface of a tooth that presented an evident cavity or softened tissue at the base of the enamel, discolouration of the enamel, or a temporary restoration (except glass ionomer). The CPI probe was used to confirm visual evidence of caries on the occlusal, buccal, and lingual surfaces.

### Unhealthy food consumption

The instrument proposed by the Brazilian Ministry of Health was used to evaluate the number of times/week each unhealthy food was consumed on a continuous scale from 0 to seven times/week. The unhealthy foods investigated were: (1) French fries, potato chips, and fried snacks; (2) hamburgers and sausages (sausage, salami, sausage ham, etc.); (3) salty crackers; (4) sweet or sandwich cookies with filling / cookies, candies, and chocolates (in bars or candy); (5) regular soft drinks consumption. Afterwards, we stratified the weekly consumption of unhealthy foods into low = up to 2 times a week (0), moderate = 2 to 4 times/week, and high = more than 4 times a week [13].

### Covariates

Sex was dichotomized into male (0) and female (1). Equalized per capita income was dichotomized into up to the poverty level (0) and above the poverty level (1) in the Brazilian context (R$ 466/month in 2018 / US$ 120.4) based on the mid-point of open-ended income [15]. The self-reported ethnic group was stratified into 0 (white) and 1 (black, brown, yellow, indigenous) [16]. The parent’s educational level was stratified into primary school grades 1–4 (0) and above grade 4 (1). Brushing teeth was stratified into up to 1 time per day (0) and two or more times per day (1), and use of dental floss 0- no 1 – yes. Water fluoridation was collected following Vigifluor research [17].

### Dental services use

The question used to collect this data was: When did your child last visit the dentist? With response options: less than 1 year; between 1 and two years; between two and three years; more than three years; never was; and doesn’t know. This variable was dichotomized into: yes (used up to three years); and no (used more than three years, never been or don’t know). This was the cutoff point established because, of those who reported using the services, none of the adolescents’ guardians reported using the services for more than three years, and only 10% of the adolescents used the services between one and three years [4].

### Theoretical framework model

The theoretical model of Sisson [18], was used to select the variables. We used variables that are related to the social determinants of oral health inequalities. Variable reflecting health-damaging choices, such as inadequate tooth brushing, unhealthy food consumption and access to oral health services, measured the cultural/behavioural explanations of Sissons´s model. Access to public supply of fluoridated water measured the contextual perspective of Sisson’s model.

### Machine learning approach

The Extreme gradient boost algorithm (Xgboost), based on sequential models of decision trees, the decision tree and the logistic regression with the Lasso penalty was used to predict untreated dental caries for adolescents. Previous research has shown that the Xgboost algorithm has the highest area under the receiver operating characteristic curve (AUC) than others [19–21].

Firstly, the dataset was split under a proportion of 75% (training set) and 25% of the testing set and then one recipe for all variables was performed, where every categorical variable was dummied, missing values omitted and normalised continuous variables were avoided oversized effects due to differences in scale. Next, we applied 5-fold cross-validation to tune hyperparameters for the training set to avoid overfitting.

### Strategy for tuning hyperparameters

The workflow was constructed, and the strategy to tune hyperparameters was 2 by 2. The sequence was: Step 1 – Tuning number of trees and learning rate; Step 2 - Tuning tree depth and minimal node size; Step 3- Tuning minimal loss reduction; Step 4 – Tuning mtry and sample size, Step 5 – tuning the learning rate and the number of trees again with all hyperparameters tuned. The select best function was used to select the best hyperparameter according to AUC values. In step 6, the Collect_metrics function to visualize the AUC, accuracy, sensitivity and, including the roc curve. After each tuned hyperparameter selected by the (AUC) for each 5-fold cross-validation model, they were tested in the test set and their predictive performance on the test set.

All of the results presented here are from the test set. Finally, to assess the predictive performance of the trained algorithm, the AUC, accuracy, sensitivity and specificity were calculated.

Furthermore, we computed the importance of each covariate in predicting our study outcomes. We used R (R Foundation for Statistical Computing, Vienna, Austria) software for our machine learning approach. We followed the STROBE guidelines for human observational studies [22] and the checklist for the artificial intelligence approach [23].

### Ethical aspects

The survey was approved under CNS resolution 466/12, CAAE 85647518.4.0000.0021. All participants provided their written consent terms, and the parents/guardians provided their written informed consent.

## Results

The prevalence of untreated dental caries was 25.3% (CI 95% 18.8–33.1). Of the 615 adolescents; the self-declared Blacks, under the poverty level, without exposition to water fluoridation, with high unhealthy food consumption, without using dental floss and with brushing habits of one or less per day had a higher prevalence of dental caries than their counterparts (Table 1).

**Table 1:** Descriptive characteristics and proportions of the Mato Grosso do Sul - Oral Health Survey (SBMS study 2018-19), for 12 year-olds and untreated caries (*n* = 615)

Individual variables	*n* = 615	% (95%CI)	Untreated caries (D ≥ 1)	
			% (95%CI)	
Ethnic Group				
Whites	270	43.9(40.0-47.9)	20.9 (14.5–29.2)	
Brown	266	43.3(39.4–47.2)	28.3 (21.2–36.7)	
Asian	24	3.9 (2.6–5.8)	23.0 (6.7–55.4)	
Blacks	32	5.3 (3.7–7.3)	57.9 (24.3–85.4)	
Indigenous	6	0.9 (0.4–2.1)	50.0 (45.3–54.7)	
Missing	17	2.8(1.7–4.4)	35.5 (17.8–57.9)	
**Sex**				
Female	315	51.2 (47.3–55.2)	24.2 (17.0-33.1)	
Male	300	48.8(44.8–52.7)	26.9 (19.6–35.5)	
**Per capita Income (equalized)**				
under the poverty level (US$ 120.4/month)	216	35.1 (31.4–39.0)	32.4 (20.3–47.5)	
above poverty level	376	61.1 (57.2–64.9)	23.3 (15.9–32.8)	
Missing	23	3.7 (2.5–5.3)	13.0 (8.3–19.9)	
**Parents’ Schooling**				
1 to 4 years	381	62.0 (58.0-65.7)	26.1 (18.9–34.8)	
above 4 years	234	38.0 (34.3–42.0)	22.6 (12.7–37.1)	
**Unhealthy foods consumption**				
Low (up to 2 times/week)	168	27.3(23.9–31.0)	9.4 (6.6–13.2)	
Moderate (2–4 times/week)	197	32.0(28.5–35.8)	31.4 (23.8–40.2)	
High (more than 4 times/week)	217	35.3(31.6–39.2)	40.4 (29.7–52.1)	
Missing	33	5.4(3.8–7.5)	60.2 (25.2–87.2)	
**Brushing**				
Twice or more per day	318	52.7 (47.7–55.6)	18.7 (12.3–27.5)	
Once per day or none	234	38.1 (34.3–42.0)	33.6 (24.4–44.3)	
Missing	63	10.2 (8.0-12.9)	38.8 (25.0-54.6)	
**Use of dental floss**				
Yes	280	55.3 (46.9–63.4)	18.6 (11.7–28.2)	
No	272	36.0 (25.7–47.8)	32.4 (23.7–42.4)	
Missing	63	8.7 (4.8–15.1)	38.8 (25.0-54.6)	
**Contextual**				
Fluoridation status				
Yes	411	66.8 (63.0-70.4)	21.6 (14.2–31.4)	
No	204	33.2 (29.6–37.0)	42.3 (35.1–49.9)	

In the Machine learning approach, the xgboost had the better performance with an AUC of 0.84, compared to 0.81 for the decision trees and 0.73 for logistic regression with the Lasso penalty algorithms. Importantly, all algorithms have demonstrated that health behaviours (use of dental floss and unhealthy food consumption) were the important variables in predicting adolescents with untreated dental caries (Table 2).

**Table 2:** Machine learning approach and metrics for adolescents. SBMS 2018/2019. (*n* = 615)

Collect metrics	xgboost		Decision trees		Logistic regression
Sensitivity	0.42(CI95% ± 0.02)		0.44(CI95% ± 0.02)		0.40(CI95% ± 0.02)
Specificity	0.92(CI95% ± 0.02)		0.88(CI95% ± 0.02)		0.80(CI95% ± 0.02)
Acuraccy	0.75(CI95% ± 0.01)		0.79(CI95% ± 0.01)		0.76(CI95% ± 0.01)
AUC	0.84 (CI95% ± 0.01)		0.81(CI95% ± 0.01)		0.73(CI95% ± 0.01)
**Three Main contributors**	importance*	**Three Main contributors**	importance*	**Three Main contributors**	importance*
Dental Floss**	0.37	Unhealthy**	0.20	Unhealthy**	0.15
Unhealthy consumption **	0.30	Whites	0.17	Dental Floss**	0.13
Whites	0.17	Dental Floss**	0.14	Fluoridation	0.09

* importance ranges from 0 to 1. The main gain in information, the more important the predictor role on the outcome ( untreated dental caries)

** Possibly modified factors to be addressed for Primary health care workers

## Discussion

This investigation showed one important finding. The xgboost algorithm should have been used and had good metrics to detect adolescents with untreated dental caries in primary healthcare settings in the Brazilian context.

This investigation has some strengths and limitations. Because this was cross-sectional data, some limitations must be pointed out. Only data from public schools were collected, which limits the study generalization for all 12-year-old adolescents at the state level [4, 13]. Concerning the representativeness of the study population, the five cities are the most representative of the four territorial macro-regions in the State and are the major affluent areas [4, 13]. As another strength, this machine learning approach would represent better the local level of data and could be applied better in this context.

It is of fundamental importance to identify adolescents with untreated dental caries to schedule an appointment in primary health care [24], especially for the most vulnerable adolescents [24]. This can help with the principle of equity in universal health coverage, giving more attention to those who need it most. The algorithm trained for the Brazilian context is easy to use and with only a few input variables, generally collected in one visit by community health workers [21], the same way to detect tooth loss in the PHC. In the present investigation an AUC of 84% was achieved, that is, getting 84 out of 100 adolescents right. This is of great value for the planning of health services throughout the Brazilian territory, and, it was a good metric obtained in this context. To the author´s knowledge, no other study has tested these algorithms to identify adolescents with untreated dental caries. The advantage of using artificial intelligence is that even without a dental consultation by a dentist in the PHC, the Family Health Strategy could use the trained algorithm. If correctly implemented, the work process has the potential to be changed in the country. In addition, the consultations could be targeted at adolescents who need care for untreated dental caries. The algorithm can function as a support for care coordination integrating the principles of the unified health system.

Furthermore, if the FHS did not have oral health teams, they could refer those to another health service, improving the FHS network system and providing better management in primary health care for adolescents [1, 2].

The implementation of algorithms in primary health care should consider the use of implementation science and its frameworks [21, 25, 26]. It is necessary to listen to the workers in the process and to assess the organizational readiness for implement change [21, 27, 28], a multilevel construct that considers the organizational readiness of primary healthcare settings to use the algorithm. Some barriers to implementation should be stated. Not every PHC unit has a computer, to collect data and process the algorithm. However, as the software used is free for machine learning, it could be implemented using online dashboards. Moreover, the acceptability, feasibility and other implementation outcomes should be tested. Future works using machine learning approaches to detect adolescents and children with untreated caries need to be further investigated, in terms of adoption, sustainability and fidelity of the intervention. Although the most important variables to predict were health behaviours (dental floss use and unhealthy food consumption) they could be modifiable factors that FHS should focus on to maximize health promotion strategies for the adolescents [21].

In conclusion, the machine learning approach performed well in predicting adolescents with untreated dental caries, using Sisson’s theoretical model. Family health teams can improve the work process and use artificial intelligence mechanisms to predict adolescents with untreated dental caries, and, in this way, schedule dental appointments for the treatment of adolescents earlier. Moreover, implementation science should be used to implement algorithms in the real world, and different implementation outcomes need to be tested before AI is used in these settings.

## Data Availability

The data that support the findings of this study are available upon request. RAB should be contacted.
